# Antithyroid drugs induced agranulocytosis and multiple myeloma: case report and general considerations

**Published:** 2013-09-25

**Authors:** R Dănciulescu Miulescu, M Carșote, R Trifănescu, D Ferechide, C Poiană

**Affiliations:** * “Carol Davila" University of Medicine and Pharmacy, Bucharest, Romania; ** “C.I. Parhon" National Institute of Endocrinology, Bucharest, Romania

**Keywords:** thiamazole, Graves’ disease, agranulocytosis, multiple myeloma

## Abstract

Antithyroid drugs as thionamides are largely used in the treatment of the thyrotoxicosis. Side effects were reported in less than 10% of the cases, especially hematological, hepatic or skin allergies. One of the most severe manifestations is agranulocytosis, probably based on an immune mechanism that is exacerbated by the presence of the thyroid autoimmune disease itself. If the presence of the severe leucopenia is actually an epiphenomenon of a preexisting hematological disturbance as multiple myeloma is debated. The myeloma may also be correlated with an autoimmune predisposition. We present the case of a 56 years old female patient diagnosed with Graves’ disease, who developed agranulocytosis after 8 months of therapy with thiamazole. Two months after antithyroid drug’s withdrawal, the granulocytes number increased and she received therapy with radioiodine. Two years later she came back for diffuse bone pain that turned out to be caused by a multiple myeloma, confirmed by bone marrow biopsy. It might be a connection between the severe form of leucopenia that the patient developed and the medullar malignancy.

## Introduction

Thiamazole is an antithyroid drug, largely used in the treatment of Graves’s disease. One side effect is granulocytopenia up to agranulocytosis, that makes necessary a periodic blood count and withdrawal of medication once it was discovered. The pathological profile of those cases with hematological disturbances is still unclear. It seems that autoimmunity, high doses of thiamazole increases the risk. Age is not a risk factor. If the chance is higher in those patients who are at risk of hematological malignancy is still debated.

## Case presentation 

A 56-year-old female patient was admitted to our hospital for 20 kg weight loss, fatigability, peripheral tremor, intolerance to heat; symptoms were presented for the last 3 months. The clinical exam revealed homogenous goiter associated with signs of hyperthyroidism (tachycardia; soft, wet skin; tremor). 

**Figure 1:**
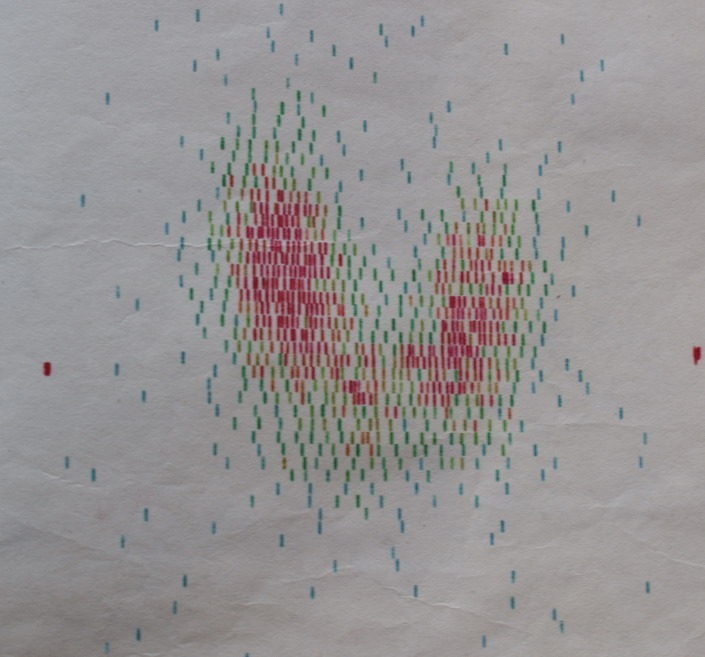
The I131 scintigraphy showing a large, homogenous thyroid gland

 The blood count and biochemical panel were normal. The hormonal profile confirmed the hyperthyroidism: suppressed thyroid-stimulating hormone (TSH), high triiodothyronine (T3)–436 ng/mL (normal range between 80 and 200 ng/dL), and high thyroxine (T4)–20.3 μg/mL (normal range between 4.5 and 13 μg/dL). Autoimmune etiology of thyrotoxicosis was suggested by elevated TPO antibodies-439 UI/mL (normal range: <36 IU/mL). The 131I scintigraphy revealed large, homogenous goiter; radioiodine uptake was high at 2 hours (29%, normal range: 12+5%) and 12% at 24 hours (normal range: 30+5%) (**Fig. 1**). Thyroid ultrasound confirmed the homogenous, hypoechoic pattern. Symptoms or signs of Graves’ ophthalmopathy were absent.

Thyrotoxicosis due to Graves’s disease was diagnosed, and treatment with antithyroid drugs, thiamazole, 40 mg per day, p.o., was started. The dose was progressively decreased to 20 mg per day. No acute adverse reaction was registered. The disease was slowly equilibrating for the next 8 months, when marked leucopenia (leucocytes–1200/mm3), but normal platelets and red cells count were found. Thiamazole was stopped and glucocorticoids (Dexamethasone) were initiated. There was persistence of thyrotoxicosis (TSH<0.03 mIU/L), requiring lithium treatment for the reducing of thyroid hormones levels. 

Two months later, the complete blood count was normal (leucocytes–5100/mm3) and lithium was stopped. Radical ablative therapy with radioiodine (5 mCi 131I) was administered; hypothyroidism was noted 2 months later, and levothyroxine 50 μg per day was recommended. The blood count showed mild anemia (hemoglobin–9.9 g/dL) and increased erythrocyte sedimentation rate (ESR) – 36 mm at 1 hour, (normal range between 1 and 25 mm at 1 hour). 

**Figure 2:**
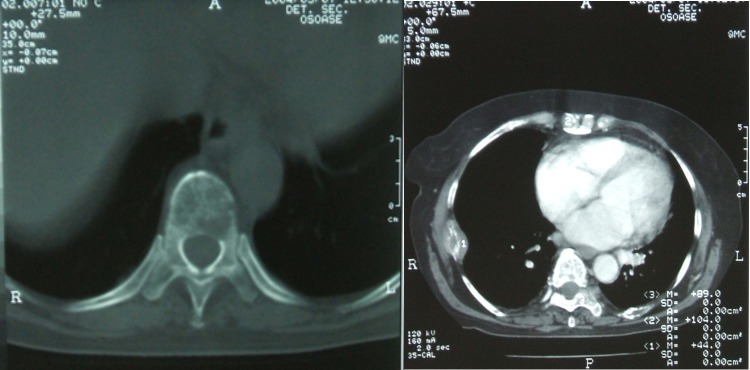
Computed tomography exam showing osteolytic lesions with broken cortical, at the level of the thoracic vertebral bodies, the right ribs and the sternum

 Two years later, the patient was admitted for diffuse bone pain, fatigability and medium efforts dyspnea. The blood count showed an aggravated anemia (hemoglobin–7.9 g/dL) and markedly increased erythrocyte sedimentation rate (ESR – 120 mm at 1 hour). The serum protein levels were increased (total serum protein–11.6 g/dL; normal range: 6.6-8.3 g/dL). The serum protein electrophoresis showed monoclonal hyper gamma-globulinemia. The thoracic-abdominal computed tomography scan revealed osteolytic lesions of 2.17/1.9 cm with broken cortical, at the level of the thoracic vertebral bodies, of 4.09/2.38 cm at the right ribs and sternum (**Fig. 2**). 

**Figure 3:**
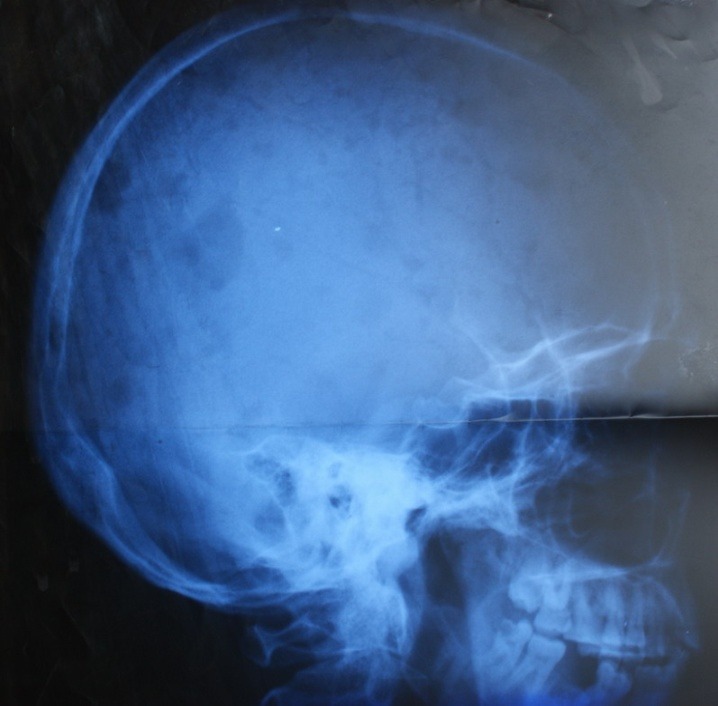
Skull X-Ray exam showing disseminated osteolytic lesions

 The skull X-Ray exam showed disseminated osteolytic lesions, with a diameter varying from a few millimeters to 2 cm (**Fig. 3**). 

The bone marrow examination revealed a highly cellular marrow, with 57-60% large plasma cells, having round nuclei, abundant, intense basophile cytoplasm. The other series, granulocytes (23%), eritroblastic (13%) and megakaryocytic lines were abnormally low, with arrested maturation. Based on these, diagnosis of multiple myeloma was established (stage II according to clinical stages of Durie and Salmon). Treatment with alkylation agent Melphalan and bisphosphonate Clodronate was started, but unfortunately the patient died 2 months later. 

**General considerations **

***Antithyroid drugs***

 The antithyroid drugs are derived from amides as thioamide (or thionamide) or derived from thiourrhea as propylthiouracil (PTU). In the first category the most known drug is thiamazole (or methimazole) and also carbimazole that is converted in vivo into the first one. Thioamides inhibit the enzyme thyroid peroxidase in the thyroid, reducing the synthesis of triiodothyronine (T3) and thyroxine (T4), and blocking the uptake of iodotyrosines from the thyroid colloid. PTU also acts by inhibiting the tetraiodothyronine 5' deiodinase that converts T4 into T3. Maximum effects occur only after a month since hormone depletion is caused by reduced synthesis, which is a slow process. Side effects of antithyroid drugs are seen in 1 up to 10% of the patients as skin eruptions as macula or papula, dermatitis, urticaria, artralgia, hepatitis, jaundice, muscle pain, thrombocytopenia, abnormal hair loss, vomiting, loss of taste. Life threatening agranulocytosis developed in 0.03% of all patients. 

***Agranulocytosis***

 Agranulocytosis is a severe type of leucopenia particularly of the neutrophils. Generally the granulocytes (a class that includes neutrophils, basophiles and eosinophils) decrease below 500 cells/mm³ of blood or less than one-sixth of the reference value of 3-10 x 103 cells/mm³. The terms of agranulocytosis, granulocytopenia and neutropenia are often used interchangeably, but agranulocytosis is the most severe form. Precisely, neutropenia describes absolute neutrophile counts less than 500 per microlitre, whereas agranulocytosis means less than 100 per microlitre. Granulocytopenia is defined by a decrease below 1500 cells per microlitre. 

 Agranulocytosis has been reported in about 0.35% up to 1.75 % of the patients treated with methimazole [**1,2**]. Granulocytopenia appears in about 2.5 % of cases [**3**]. The risk of agranulocytosis is increased when high thiamazole doses are used. In a retrospective study, agranulocytosis was observed in 4.1% of patients treated with at least 20 mg per day of methimazole, compared with only 0.31% from the patients with smaller doses [**4**]. According to the same authors, age does not seem to be a risk factor [**5**] but former studies did not reach to the same conclusion [**6**]. 

 Usually, the reaction develops after 2 up to 12 weeks of treatment; however, in our case, leucopenia developed after 8 months treatment, despite periodic complete blood counts. It frequently has acute onset, but cases have been described in which it followed after granulocytopenia [**7**]. In our case there was an asymptomatic form. Agranulocytosis was found even in patients who previously tolerated the antithyroid drugs, after several exposures. Multiple exposures may represent a risk factor for developing hematological disturbances [**2,3**]. Cross reactions between the different thioamides may appear, so replacing one drug with another is not recommended [**8**]. 

 The most common manifestation of leukopenia refers to febrile infections, frequently upper respiratory tract infections as angina and gingivitis [**3,9**].The most severe form is sepsis [**1**]. In such cases, the bone marrow biopsy usually shows decrease in the numbers of granulocytes and their precursors, or cellular hyperplasia. Maturation arrest in the myeloid line is common and sometimes it is the only finding [**3,10**]. The cost effectiveness of serial blood count in order to detect the granulocytes abnormalities is still a matter of debate. Although not widely recommended, it has the advantage of identifying patients before the clinical manifestations occur, like in our case [**1**]. Another still open question is the usefulness of the therapy with granulocyte - colony stimulating factor (G-CSF), which is generally recommended for patients with drug-induced granulocytopenia. A Japanese study on 109 patients with thiamazole induced granulocytopenia did not found benefits in treating symptomatic patients with very low granulocyte counts, although in less severe cases the recovery time was shortened. Prolonged treatment in unresponsive patients is not effective [**11**]. There has been suggested that measuring the granulocytes 4 hours after the injection with G-CSF may detect the patients in which this treatment is effective [**12**]. The effect of treatment with Dexamethasone has not been certainly proved [**1**]. 

 Older studies showed a mortality of up to 18% for agranulocytosis [**9**], but, during the last years, because of better treatment of infections and maybe due to the use of G-CSF, the fatal outcome appears in less than 5% of cases [**13**]. 

Aplastic anemia is a rare life threatening complication of the antithyroid medication that develops only in association with agranulocytosis. It usually appears soon after the beginning of the treatment and resolves after drug’s withdrawal [**14**]. Bone marrow is hypocellular, in some cases with plasmacytosis, suggesting an immunogenic cause [**15**]. The treatment is supportive, but cases responding favorably to G-CSF have been described [**8**]. 

***The agranulocytosis and autoimmunity ***

 The etiopathogeny of agranulocytosis is still unclear. Immunologic mechanism has been incriminated, because anti-granulocyte antibodies have been identified in certain cases, but other mechanisms are probably involved [1,2]. Methimazole does not cause a decrease in the levels of natural G-CSF [**10**]. 

 Methimazole has also other autoimmune side effects. In a small prospective study, 3 out of 33 patients on thiamazole developed antineutrophil cytoplasmic antibody (ANCA) and other 2 had ANCA-positive vasculitis, with renal involvement [**16**]. One case of central nervous system vasculitis has also been described [**17**]. These autoimmune syndromes seem to disappear after the discontinuation of the drug [16,17]. Methimazole has been involved in the development of insulin antibodies [**18**]. Other rare complications, possible with similar mechanism including pancreatitis and parotiditis have been described [**19**]. 

***Multiple myeloma and autoimmunity***

 Multiple myeloma is a malignancy of plasma cells, which can range from monoclonal gammopathy of unknown significance (MGUS) to plasma cell leukemia. It represents about 15% of all lymphatic hematopoietic neoplasia, with a higher incidence in black men [**20**]. The incidence increases with age. 

 The disease is characterized by an over-production of monoclonal paraproteins, leading to hyper viscosity, amyloidosis, renal failure and disrupting the normal immune response. Because of proliferation of plasma cells in the bone marrow, patients develop osteolytic lesions and hypercalcemia. Our patient had bone lesions, but no hypercalcemia. Even if new therapy protocols, including autologous stem-cell transplantation have been developed and are effective, the disease has a median survival period after the diagnosis of about 3 years (higher in younger patients) [**2**]. 

 The etiology of multiple myeloma in unknown, but an association with chronic stimulation of the immune system, like in autoimmune diseases has been described [**21**]. The data concerning multiple myeloma and thyroid pathology is somewhat controversial. In a retrospective study on 73 cases of patients with multiple myeloma compared with gender and age controls showed a higher incidence of autoimmune thyroid disease. The authors conclude that patients with thyroid pathology have an odds ratio of 2.41 for developing multiple myeloma, compared with healthy controls [**20**]. Also, a familial history of thyroid disease is associated with increased risk of multiple myeloma. This finding was not confirmed by a case-control study performed on 179 female patients with multiple myeloma which did not found any correlation between thyroid medication and the hematological disease [**22**]. Also, a large Swedish population based study (on over 8000 patients with multiple myeloma) did not show a strong correlation with personal or familial autoimmune diseases [**23**]. 

## Conclusions

We present this case as an argument for the periodical check up of the blood count during the treatment with antithyroid drugs in order to detect anomalies as leukopenia and even worse agranulocytosis. It is difficult to express any certain correlation between the autoimmune thyroid disease as Graves’s disease and the risk of hematological disturbances. It is possible that the patients who develop agranulocytosis actually have more severe marrow diseases as malignancy and the drop of granulocytes is in fact the exposed part of an iceberg. Further serial investigations should be performed. 
